# An Overview of Long COVID Support Services in Australia and International Clinical Guidelines, With a Proposed Care Model in a Global Context

**DOI:** 10.3389/phrs.2023.1606084

**Published:** 2023-09-22

**Authors:** Shiqi Luo, Zhen Zheng, Stephen Richard Bird, Magdalena Plebanski, Bernardo Figueiredo, Rebecca Jessup, Wanda Stelmach, Jennifer A. Robinson, Sophia Xenos, Micheal Olasoji, Dawn Wong Lit Wan, Jacob Sheahan, Catherine Itsiopoulos

**Affiliations:** ^1^ School of Health and Biomedical Sciences, STEM College, RMIT University, Bundoora, VIC, Australia; ^2^ Department of Health and Biostatistics, Swinburne University of Technology, Hawthorn, VIC, Australia; ^3^ School of Economics, Finance and Marketing, College of Business, RMIT University, Melbourne, VIC, Australia; ^4^ Northern Health Australia, Epping, VIC, Australia; ^5^ School of Media and Communication, College of Design and Social Context, RMIT University, Melbourne, VIC, Australia; ^6^ Institute of Health and Wellbeing, Federation University Australia, Ballarat, VIC, Australia; ^7^ Institute for Design Informatics, University of Edinburgh, Edinburgh, United Kingdom

**Keywords:** long COVID, care model, guidelines, long COVID services, post COVID-19 condition, multidisciplinary

## Abstract

**Objective:** To identify gaps among Australian Long COVID support services and guidelines alongside recommendations for future health programs.

**Methods:** Electronic databases and seven government health websites were searched for Long COVID-specific programs or clinics available in Australia as well as international and Australian management guidelines.

**Results:** Five Long COVID specific guidelines and sixteen Australian services were reviewed. The majority of Australian services provided multidisciplinary rehabilitation programs with service models generally consistent with international and national guidelines. Most services included physiotherapists and psychologists. While early investigation at week 4 after contraction of COVID-19 is recommended by the Australian, UK and US guidelines, this was not consistently implemented.

**Conclusion:** Besides Long COVID clinics, future solutions should focus on early identification that can be delivered by General Practitioners and all credentialed allied health professions. Study findings highlight an urgent need for innovative care models that address individual patient needs at an affordable cost. We propose a model that focuses on patient-led self-care with further enhancement via multi-disciplinary care tools.

## Introduction

Since its outbreak in January 2020, the COVID-19 pandemic has resulted in an estimated 642 million cases and over 6.6 million deaths globally [1, 2]. In Australia, more than 11 million cases have been confirmed and over 19,000 deaths recorded [3].

While the majority of those infected recover fully, up to 40% of individuals may experience lingering symptoms including fatigue, shortness of breath, persistent cough, joint pain, brain fog, cognitive dysfunction, anxiety, depression, loss of smell or taste, and insomnia [4]. These symptoms may fluctuate, relapse over time and persist for several months. When symptoms persist beyond 12 weeks [4] the condition is deemed chronic and termed Long COVID or Post COVID-19 condition by the World Health Organization (WHO) [5]. In addition to these terms, several terms have been referenced including “long haulers,” “post-acute COVID-19,” “post COVID-19 syndrome” and “post-acute sequelae of the severe acute respiratory syndrome coronavirus 2 (SARS-CoV-2) infection” [6, 7]. For the purposes of this review, we have adopted the term “Long COVID” to describe this condition.

Long COVID poses a significant challenge as an emerging health condition for several reasons. Firstly, the underlying pathology of this condition is still being examined and effective treatments are not yet available [8]. Secondly, the emergence of Long COVID is occurring amidst an ongoing struggle of healthcare systems worldwide to manage the demands associated with the continued presence of COVID-19 [8]. Thirdly, Long COVID is a complex and heterogeneous health condition, with more than 50 different clinical symptoms affecting 10 different body systems [9]. [10], and its duration is still uncertain. Long COVID is a “major health challenge for the coming couple of years at least” [10].

Health authorities and services have responded to the needs of those affected by Long COVID by developing guidelines and providing services to assist recovery. To evaluate if the current status of services offered meets the needs of people affected by Long COVID, this review aims to present:

(i) A critical summary of national and international Long COVID guidelines;
(ii) A summary of the Long COVID support services currently being delivered in Australia;
(iii) An evaluation on whether the characteristics of these services are in accordance with national/international guides and identification of gaps; and
(iv) Recommend a care model for future services.

## Methods

### Search Strategy

To identify Long COVID guidelines, the following databases and websites were searched; Google Scholar, PDQ-Evidence, WHO, Centers for Disease Control and Prevention (CDC), National Institutes of Health (NIH), The Australian Royal Australian College of General Practitioners (RACGP), The National Institute for Health and Care Excellence (NICE), Public Health Agency of Canada and Ministry of Health NZ. Search terms included “Long COVID guidelines,” “Post COVID conditions guidelines” AND “Post COVID-19 condition” between 10 August to 23 September 2022 to obtain articles from 2020 onwards. Follow up searches were conducted on 26 January 2023 and 22 March 2023, respectively. The Guidelines International Network (GIN) repository was searched using the same search terms on 12 July 2023.

To identify Long COVID services in Australia, Google searches were undertaken using the terms “Long COVID service OR clinic Australia,” “Long COVID service OR clinic Victoria,” “Long COVID service OR clinic New South Wales,” “Long COVID service OR clinic Australian Capital Territory,” “Long COVID service OR clinic Queensland,” “Long COVID service OR clinic South Australia,” “Long COVID service OR clinic Western Australia,” “Long COVID service OR clinic Tasmania.” “Long COVID service OR clinic Northern Territory,” “Post covid service OR clinic Australia” between 10 August and 23 September 2022. Follow up searches were conducted on 26 January 2023 and 22 March 2023, respectively.

### Selection Criteria

The following inclusion criteria to guidelines for Long COVID were utilized: 1) provide comprehensive information such as care principles, care models, and patient education on Long COVID and 2) developed in English-speaking countries. Clinical guidelines were excluded if they did not state the process of the guideline development as indicated in AGREE II assessment tool [11].

In regard to Long COVID services, only those: 1) Were located in Australia; 2) Offered Long COVID-specific services; 3) Provided information on the services offered.; 4) The information was available to the public were included.

### Data Extraction and Synthesis

**FIGURE 1:**
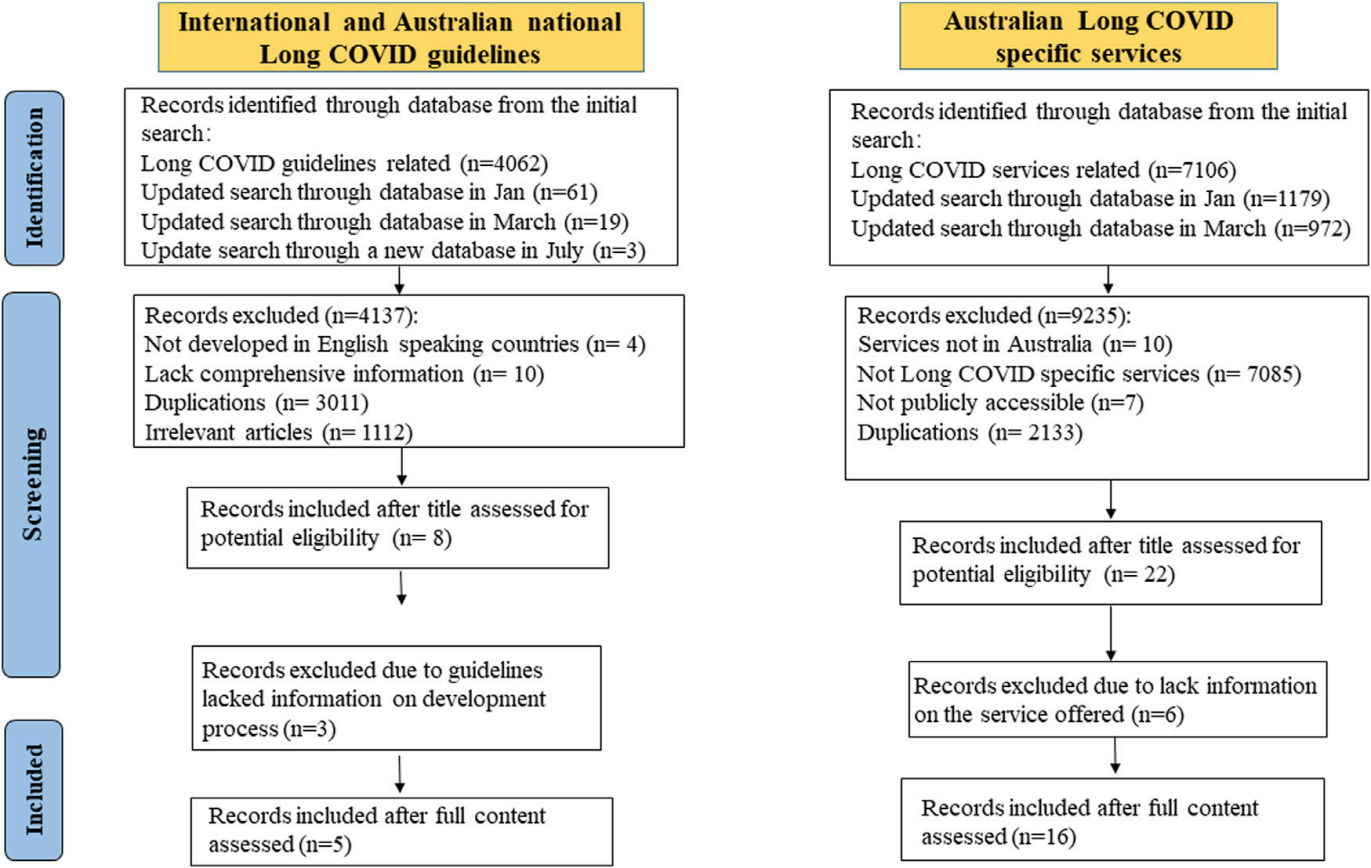
A flow chart of information selections (Australia, 2023).

Data selection was conducted by one author (SL) and extracted and verified by two authors (SL and ZZ). The key data extraction items were developed from our aims and previewing of the content, then presented in tables or figures in a narrative format (Supplementary Appendix S1 and Figure 1).

### Quality Assessment

Guidelines were evaluated by two independent appraisers (SL and ZZ) using the AGREE II assessment tool [11], covering six domains (scope and purpose, stakeholder involvement, rigor of development, clarity of presentation, applicability, and editorial independence) with 23 items rated on a 7-point scale (1 = strongly disagree; 7 = strongly agree). Domain scores were calculated following the recommended formula [11]. Guidelines scored below 50% were rated low-quality, 50%–70% moderate, and >70% high-quality [11].

### Consumer Engagement

Three individuals with lived experience of COVID-19 or Long COVID and who had sought medical advice for management reviewed the manuscript and provided feedback.

## Results

The search identified 8 Long COVID specific guidelines and 16 Long COVID specific services in Australia. Three out of eight guidelines were excluded (two from Canada and one from Australia) as they summarised existing guidelines without providing information about their development process. Five guidelines and 16 associated services were included in this review. Figure 1 illustrates the study selection process.

### A Critical Summary of National and International Guidelines

Table 1 is a summary of the guideline characteristics, including the definition of Long COVID, suggested review period, multidisciplinary team and patient education.

**TABLE 1:** Characteristics of the selected Long COVID guidelines (Australia, 2023).

References	Definition of long COVID	Suggested review/ referral period	Multidisciplinary team	Patient education
[5] WHO	• The continuation symptoms 3 months after the SARS-CoV-2 infection	After 12 weeks	Physicians, Nurses, Pharmacists Physiotherapists, OTs, Psychologists, Speech and language therapists, Social workers, Dieticians, Community healthcare workers	Recommendations for managing key symptoms includ**e breathing techniques, pacing, stress management**
	• Symptoms that last for at least 2 months			
	• Cannot be explained by an alternative diagnosis			
[12] Australia	• The continuation symptoms 3 months after the SARS-CoV-2 infection	Early review after 4 weeks	General Practitioners, Physiotherapists, Exercise physiologists, OTs, Dietitians, Speech pathologists, Psychologists, Environmental physicians, Rehabilitation medicine physicians, Geriatric medicine physicians	Guide available for **self-managing** post COVID-19 conditions, Encourage patients to fill in a symptom diary every few days
	• Symptoms that last for at least 2 months			
	• Cannot be explained by an alternative diagnosis			
[13] United States	• A failure to return to normal health after having acute SARS-CoV-2 infection	Initial 4–12 weeks after acute COVID-19	Physiotherapists, OTs, Speech therapists, Neurologic rehabilitation specialists, Nutritionists, Sleep specialists	Simple resources available for **self-management**, Encourages patient symptom diaries and calendars, Provide links to support groups
	• the emergence of new or recurring symptoms or the revealing of an existing condition after the acute COVID-19 symptoms have gone			
	• Recovery does not happen within 4 weeks after the acute phase			
[14] United Kingdom	• The continuation symptoms 3 months after the SARS-CoV-2 infection	Early review after 4 weeks	Physiotherapists, Psychologists, Psychiatrists, OTs Specialists	New or ongoing symptoms after acute COVID-19, Expectations for recovery, Recommendations for **self-management**, When to seek help from healthcare professional, Symptom diaries and symptom tracking apps
	• Symptoms that last for at least 2 months			
	• Cannot be explained by an alternative diagnosis			
[15] New Zealand	• The continuation symptoms 3 months after the SARS-CoV-2 infection	After 12 weeks	Clinical exercise physiologist, Physiotherapist, OT, Dietician, Psychologist, Social worker, Speech therapist,	Links and helpline numbers for **mental health** support, Links for Long COVID related symptom management, Peer support, Resources for children and young people with Long COVID, Care plans and action plans
	• Symptoms that last for at least 2 months			
	• Cannot be explained by an alternative diagnosis			

Five international guidelines were developed by the WHO [5], the United States (US) Centres for disease control and prevention (CDC) [13], a collation of the United Kingdom (UK) guidelines [The National Institute for Health and Care Excellence (NICE); the Scottish Intercollegiate Guidelines Network (SIGN), and the Royal College of General Practitioners (RCGP)] [14, 16], the Australian Royal Australian College of General Practitioners (RACGP) [12] and New Zealand Ministry of Health [15]. The WHO guidelines serve as the cornerstone for post-COVID care, while the Australian, United Kingdom, United States, and New Zealand guidelines complement and provide detailed information on patient care. While no guidelines met our selection criteria from the Public Health Agency of Canada, two excellent patient resources on Long COVID recovery care were provided [17–19].

The definition of Long COVID used in the guidelines have similarities and differences. Four guidelines, including WHO, Australian, New Zealand and UK guidelines define Long COVID as post COVID-19 condition that occurs in individuals with a history of probable or confirmed SARS-CoV-2 infection 3 months from the onset of COVID-19 with symptoms that last for at least 2 months and cannot be explained by an alternative diagnosis [5, 12, 14, 16]. Common symptoms include fatigue, persistent cough, shortness of breath, cognitive dysfunction which generally have an impact on everyday functioning. Symptoms may be new onset, following initial recovery from an acute COVID-19 episode, or persist from the initial illness. Symptoms may also fluctuate or relapse over time [5, 12, 14, 16] A separate definition is given for children, which is not included here as it is beyond the scope of this review [5].

In contrast, the US guidelines use both the term “Post-COVID Conditions” and “Long COVID.” They define Long COVID as signs, symptoms, and conditions that continue or develop 4 weeks or more after the acute phase of COVID-19 infection [13]. Moreover, the US guidelines are the sole source that consider exacerbation of the pre-existing symptoms as part of Long COVID presentation [13].

The US guidelines have assigned the code “**U09.9 Post COVID-19 condition, unspecified”** in the International Classification of Disease, 10th Revision (ICD-10) activated by WHO to allow the establishment of a link with COVID-19. The code is separate from cases with acute COVID-19 [13]. Similarly, the New Zealand guidelines also applied an ICD code for Long COVID [15].

All guidelines stress the importance of a person-centered approach and multidisciplinary interventions that address various physical, cognitive, psychological, and psychiatric symptoms, as well as functional disabilities, alongside screening for red flags [5, 13, 14, 16]. Patient education is emphasized across all guidelines, with a focus on mental health, symptom management, and keeping a symptom diary. Importantly, all guidelines lack step-by-step approaches to skills training and interactive or visual patient education materials [5, 12–14, 16].

The UK and US guidelines recommend early investigation within 4 weeks or between 4 and 12 weeks of contracting COVID, rather than waiting for 12 weeks as implied in the WHO guidelines [5, 13, 14, 16] The Australian and UK guidelines also suggest early review after the acute phase of COVID-19 and recommend patient assessment based on various factors such as the severity and duration of symptoms, mental health and worsening of pre-existing conditions [12, 14, 16].

In addition, what sets the US guidelines apart from other guidelines is their incorporation of trauma-informed approaches, which are based on six key principles (Supplementary Appendix S2). These principles include creating a safe environment, training staff to be trauma-informed, providing peer support, involving patients in organizational planning, empowering patients to make choices, and addressing cultural, historical, and gender-related issues [13].

Furthermore, although the New Zealand guidelines may not include specific details regarding interventions and referral timelines, they do provide recommendations for a variety of population groups, including children, the elderly, indigenous peoples, and individuals with disabilities. In addition, they address vocational rehabilitation for working individuals, particularly women. This work-related specific rehabilitation has not been mentioned in other guidelines (Supplementary Appendix S2) [15].

### Quality Appraisal of the Guidelines

Table 2 presents the AGREE II scores of included guidelines. One of five (25%) guidelines (UK) was high-quality, three (75%) (US, WHO, New Zealand) were moderate-quality, and the remaining one (Australia) was low-quality.

**TABLE 2:** Methodological quality of each guideline appraised by the AGREE II instrument (Australia, 2023).

References	Scope and purpose (%)	Stakeholder involvement (%)	Rigor of development (%)	Clarity of presentation (%)	Applicability (%)	Editorial independence (%)	Agree II score (mean)	Overall quality	Overall assessment
[5] WHO	91.7	58.3	84.4	100	20.8	29.2	64.1	M	83.3
[12] Australia	80.6	36.1	15.6	91.7	41.7	0	44.3	L	50
[13] United States	88.9	58.3	34.4	94.4	45.8	0	53.6	M	75
[14] United Kingdom	97.2	97.2	92.7	97.2	72.9	87.5	90.8	H	100
[15] New Zealand	97.2	55.6	30.2	86.1	33.3	0	50.4	M	66.7

L, low quality; M, moderate quality; H, high quality.

All guidelines scored high in the domains of Scope and Purposes, and Clarity of Presentation, but poorly in Applicability, particularly lacking information on auditing criteria and facilitators/barriers to recommendation implementation, except for the UK guidelines. Editorial Independence was also a weak area, with four guidelines (Australia, WHO, US, New Zealand) failing to declare the funding body, or disclose panel members’ conflict interests. The quality of Stakeholder Involvement varied. Only two (WHO and United Kingdom) clearly stated they included Long COVID/COVID-19 survivors in the advisory committee, and one (United Kingdom) outlined the composition of the development panel. As for the Rigor of Development, three guidelines (Australia, New Zealand, United States) did not explain clearly the systematic search, evidence selection, external review, and update plans. Overall, the guidelines’ quality ranged from moderate to high.

### Long COVID Services in Australia

Among the identified services, nine are located in Victoria (VIC), two in Queensland (QLD), and one each in New South Wales (NSW), Australian Capital Territory (ACT), South Australia (SA), Western Australia (WA), and Tasmania (TAS), respectively. Unfortunately, no Long COVID service could be identified in the Northern Territory. In our 2nd updated search conducted in March 2023, a few new GP-led or allied health-led clinics designed for Long COVID management were identified in NSW [20–22] and QLD [23, 24]. However, we did not incorporate them into this review because they did not provide detailed information on the services they offer, and the health professionals involved. Table 3 provides a summary of all 16 services, including three Victorian services that have been terminated due to a lack of funding.

**TABLE 3:** Content, delivery and targeted population of long COVID service in Australia (*n* = 16) (Australia, 2023).

Service name	Setting/Organization/State	Clientele	Program delivery	Delivering method	Goal/status	References
The Victorian Rehabilitation Centre Long- COVID Outpatient Program	Hospital/The Victorian Rehabilitation Centre/VIC	A. Patients with COVID-19 symptoms lasted for ≥12 weeks after the initial diagnosis	Exercise sessions Energy conservation fatigue management and goal setting Stress management and mindfulness sessions Nutrition and lifestyle consultations	General Practitioner (GP) referral Preliminary medical screening (Telehealth) Face to face outcome measure	Ongoing service to support Long COVID patients return to activity	[25]
		B. The symptoms cannot be explained by an alternative diagnosis				
Long-COVID Rehabilitation Service (ReCOVery) (Terminated due to lack of funding)	Hospital/Austin Health/VIC	A. Patients ≥18 years and have COVID-19 for ≥12 weeks	Comprehensive assessment Individual care plans Patient education Care coordination	GP/specialist referral Initial triage assessment (SMS) Goal setting Individual discipline assessment and intervention (face to face)	Terminated due to lack of funding	[26]
		B. The new and ongoing symptoms are not contributed by pre-existing issues				
		C. Within the Austin Health catchment area				
		D. Not an aged care resident				
The Royal Melbourne Hospital- Post COVID Clinic	Clinic/The Royal Melbourne Hospital (RMH)/VIC	A. Patients had confirmed COVID-19 infection	Medical evaluation Diagnosis, prognosis, advice Initial assessment Comprehensive care plan development Allied health intervention	GP/medical specialist referral Telehealth is possible for patients outside RMH catchment Face to face therapy	Management of ongoing symptoms post the infectious status	[27]
		B. Within RMH catchment area or had care at RMH				
		C. Ongoing symptoms >6 weeks				
		D. Not acutely unwell				
The Royal Melbourne Hospital- ReCov Service	Clinic/RMH/VIC	A. Any RMH patients who have ongoing symptoms due to COVID.	Clinical and neuro psychology Social work Nutrition OT Music therapy Physiotherapy Exercise physiology Rehabilitation medicine	Collaborate with RMH Post-Covid clinic	Management of ongoing symptoms post the infectious status	[28]
		B. Can actively participate in therapy				
		C. Symptoms are not caused by other pre-existing conditions				
		D. Not undergoing therapies elsewhere for symptoms management				
Northern Health Post COVID-19 Clinic (Terminated due to lack of funding)	Clinic/Northern Health/VIC	Patients who had COVID-19 and are experiencing:	Not provided	GP Referrals	Terminated due to lack of funding	[29]
		A. Respiratory weakness				
		B. Decreased physical capacity				
		C. Reduced independence or function in daily activities				
		D. Changes in mental health				
		E. Cognition and memory issues				
		F. Sleep disturbances				
Post COVID Follow Up Alfred Health	Hospital/Alfred health/VIC	A. Patients who had acute symptoms 4–12 weeks ago	Pulmonary rehabilitation Cognitive rehabilitation Medical specialty Allied healthcare coordination Mental care plans through GP Self-management peer support	Gp referral/follow-up post discharged Alfred health patients Telephone assessment Rehabilitation	Provide support for symptoms management and gradual return to work and daily life	[30]
		B. Or 6–8 weeks post discharge follow up				
		C. Ongoing symptoms or limitations				
Geelong Long-COVID clinic	Community based clinic/VIC	A. Patients who experience symptoms >12 weeks after the initial diagnosis	Comprehensive assessment Medical tests Refer to Long COVID clinics in public hospitals Rehabilitation	Face-to face assessment Telehealth follow up	Rule out red flags and refer to appropriate service	[31]
Epworth Long -COVID- rehabilitation and mental health programs	Private hospital/Epworth rehabilitation/VIC	Epworth or non-Epworth patients with Long COVID symptoms such as shortness of breath or trouble breathing, chest pain, a persistent cough, heart problems, loss or changes in sense of smell and taste, brain fog, etc.	Cardiac rehabilitation Pulmonary rehabilitation Neurological rehabilitation Reconditioning Olfactory impairment clinic (Loss of taste or smell) Mental healthcare through Epworth Clinic	GP/specialist referral Telehealth assessment Face to face intervention	Help patients to manage and recover from a range of Long COVID symptoms	[32]
Community Health-Post COVID-19 Recovery Program Peninsula Health (Terminated due to lack of funding)	Outpatient clinic/Peninsula Health/VIC	A. Patients who are >18 years	Initial consultation and assessment Multidisciplinary intervention Care plan development Eight weeks program with follow up consultation at 3, 6 and 12 months	GP/self-referral Telehealth Face to face interventions	Terminated due to lack of funding	[33]
		B. Two weeks post a positive diagnosis of COVID-19				
		C. Within Peninsula Health catchment areas				
		D. Clinically stable				
		E. Able to consent to referral				
Post Covid Recovery Clinic- Canberra Health Service	University clinic/Canberra Health/ACT	A. Patients who are >16 years	Not provided	Gp/specialist referral Initial assessment (phone) Therapy at University of Canberra Hospital (in person) See healthcare providers in the community	Collaborate with GPs for symptoms management and help patients to achieve recovery goals	[34]
		B. ACT residents				
		C. Having ongoing symptoms >3 months				
		D. Having rehabilitation goals				
		E. Medically stable				
		F. Willing to join this rehab program				
The St Vincent hospital Sydney Long- COVID Outpatient Clinic	Clinic/The St Vincent Hospital Sydney/NSW	A. Patients who are >16 years	Assessing observations Lung function test Respiratory physician review HOPE Survey (rehabilitation screening tool) review Developing management plan	GP referral HOPE Survey prior to the appointment (SMS) Face-to face appointment	Help patients to manage and reduce long-term symptoms and assist them back to work or school	[35]
		B. Having significant symptoms ≥4 weeks from the date of positive COVID-19 diagnosis				
		C. Living in Sydney				
Long-COVID Assessment Clinic-Royal Adelaide Hospital	Clinic/Royal Adelaide Hospital/SA	A. Suspected Long COVID syndrome >12 weeks	Assessment Referring to relevant allied health or specialist services Care plan development	GP/specialist referral Initial assessment (via phone) Face to face clinic appointment	Provide advice for recovery	[36]
		B. Persistent and significant symptoms for ≥8 weeks				
		C. No urgent/red flag symptoms				
Long-COVID (post COVID) clinic	Clinic/The Wesley Hospital/QLD	A. Patients referred internally by The Wesley Hospital specialists	Assisting rehabilitation goals Allied health therapies Cardiac, respiratory, musculoskeletal rehabilitation General reconditioning	Internal referral	Provide support for reconditioning and set rehabilitation goals	[37]
The iHealth Care Long COVID clinic	Clinic/iHealth Centre Indooroopilly/QLD	A. Patients who are aged >16 years	Medical assessment, Standard biometric measurements, Management plan, Allied heath therapies, Ongoing reviews	Telehealth or face to face assessment In person treatments Reviews via video consultation or in person	Support and assist with recovery	[38]
		B. A confirmed SARS-CoV-2 viral infection (positive RAT or PCR)				
		C. Ongoing symptoms for ≥4 weeks since the initial diagnosis				
East Metropolitan Health Service (EMHS) post COVID-19 clinic	Clinic/Bentley Health Service/WA	A. Patients who are aged >16 years	Further assessment Triage Patient education	Telehealth assessment Follow-up if necessary	Help patients to be referred to appropriate services and symptoms management education	[39]
		B. Live within the EMHS metropolitan catchment				
		C. ≥ 12 weeks from the date of SARS-CoV-2 diagnosis				
		D. Have ongoing non-urgent significant symptoms				
		E. GP screens show not for direct referral to sub-specialty medical review or Community Rehabilitation				
Post COVID-19 Navigation Service	Unknown/The Department of Health/TAS	A. Patients who are >16 years	Allied health service Physiotherapy Social services	GP referral	Help patients to improve function	[40]
		B. Being a Tasmanian resident				
		C. Ongoing symptoms 12 weeks after the initial diagnosis of COVID-19				
		D. Symptoms affect physical and mental function				

Most of the active services are publicly funded, with no extra charges to patients, while three are privately funded using a mixed funding with Medicare payment and gap payments by patients either personally or through private insurance. Fourteen services require a referral from general practitioners (GP) or other medical specialties and adopt a hybrid approach of in-person and remote models. Four services include follow-up for hospital admitted patients, while one service accepts inpatient referral only, and the remaining nine services are designed for patients living with the condition in the community.

The majority of services did not specify the age of the target population, so we assumed they accept people of all ages. Several services (in WA, ACT, NSW, QLD and TAS) provide support to patients as young as 17 years old. While older people can access these services, there are no specific services for either people over 65 or children as these populations may require different support [8, 41]. Two Victorian services specifically exclude aged care residents. Furthermore, no service is available specifically for people with mental or physical disabilities, who are already disadvantaged due to their increased vulnerability to Long COVID [8].

In addition, the majority of the Long COVID services focus on providing rehabilitation programs to address specific signs and symptoms of Long COVID, such as fatigue and shortness of breath. The common features of these services include: 1) support for the delivery of rehabilitation services; 2) multidisciplinary rehabilitation; 3) continuity and coordination of care teams; 4) hybrid care delivery methods.

Based on the service information provided by the Long COVID clinics, we calculated the percentage distribution of multidisciplinary management team members across the identified services (Supplementary Appendix S3). Physiotherapists and Psychologists are the most commonly included professionals (63% each), whereas Neurologists, Liaison Psychiatrists, and Pain Specialists are less common. Surprisingly, only 19% of multidisciplinary care teams included GPs (if we exclude referral from GP).

**FIGURE 2:**
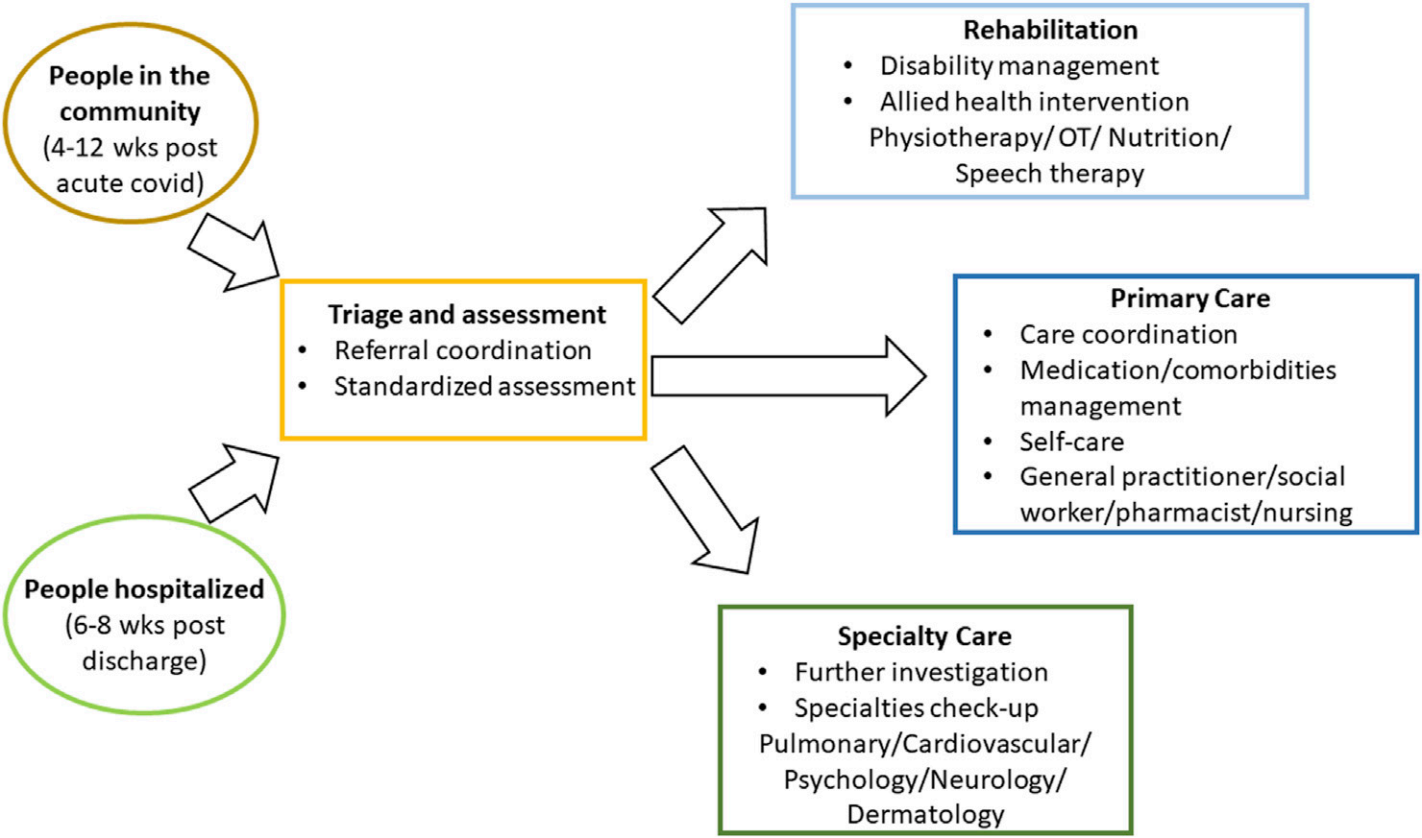
A proposed care pathway for Long COVID by a Canadian study (modified with permission [9]) (Canada, 2021).

Moreover, out of all 16 services, the Post-COVID Follow Up Service provided by Alfred Health is the only one that has explicitly described the rationale for their service design. Their approach is based on a systematic review of care models for Long COVID conducted by a group of Canadian researchers [9]. Figure 2 illustrates the four key elements of their approach, with each element working as a network to prioritize the needs of each individual living with Long COVID. The coordination unit receives referrals from both hospitalized and community-based patients and assesses and triages cases according to individual needs. Some cases are referred to multidisciplinary rehabilitation teams, medical specialty clinics (such as pulmonary, cardiovascular, psychology/psychiatry, neurology, etc.) for advanced testing and diagnoses, and others to primary care teams for screening and support. The goal is to achieve positive outcomes for patients and assisting them back to work and daily life [9].

### Gaps Between Service and Guidelines

Service models in Australia are generally align with the international and national guidelines, consisting of 1) assessment of red flags, 2) support for the delivery of rehabilitation services; 3) multidisciplinary rehabilitation; 4) coordinated care and 5) a hybrid care delivery method. It is not clear how recommendations in the guidelines are carried out in clinical practice, including assessment, patient-centred care, shared-decision making and patient education. Even though the Australian, UK and US guidelines have developed patient resources for education and self-care strategies [12–14, 16], patient education and empowerment are not clearly described in any of the identified Long COVID services.

In addition, early investigation and review of individuals who have Long COVID or who could potentially develop it are recommended by the United States, Australia and United Kingdom. However, it is unclear how this recommendation could be implemented in clinical practice.

### Considerations for Future Care Models

Based on the gaps between guidelines and current service models, future care models must consider the following aspects:

1) **Early Investigation**: When symptoms are persistent more than 4 weeks from the time of contracting virus, it is crucial to initiate investigations as this reduces the risk of developing persistent illness [13].
2) **Self-Care Engagement**: Self-care is a crucial aspect of the recovery process [12, 42]. Emphasising the importance of self-care practices empowers patients to take charge of their wellbeing and supports the healing process.
3) **Seek Professional Assistance**: If symptoms persist beyond 8 weeks, it is essential for individuals with Long COVID to seek prompt assistance from their general practitioners and specialists. This step is vital as it allows for thorough evaluation and, if deemed appropriate, referral to specialised Long COVID clinics [12, 14].

## Discussion

Our review identifies five international guidelines, with four rated as moderate to high quality and one as low quality. WHO guidelines are the cornerstone of the definition and care for Long COVID whereas the Australian, UK, US, and New Zealand guidelines complement it on patient care. Long COVID is defined as signs and symptoms persisting 4 week (United States) or 12 weeks (WHO, United Kingdom, Australian and New Zealand) after the initial confirmed or probable infection of SARS-CoV-2. All guidelines promote the importance of screening for red flags, a person-centered approach, self-care and multidisciplinary interventions. Both US and New Zealand guidelines formally recognized Long COVID through using an ICD code. The latter also acknowledges the impact of Long COVID on work capacity and recommends vocational rehabilitation.

We have identified limited Long COVID services available in Australia, with 9 out of 16 clinics in Victoria, the state that had the earliest outbreaks of COVID-19 in Australia. Among the 16 services, three have since been terminated due to lack of funding. Most services promote multidisciplinary rehabilitation programs, which are often costly to operate and can only accommodate a small number of patients. This may explain why it has not been possible to sustain some service provision. However, considering the intermittent trend of worldwide COVID-19 cases [43], introducing more or reactivating of the long covid services will be necessary.

The significant gaps identified are the early identification and referral of individuals with Long COVID at 4 weeks after initial contraction of SARS-CoV-2 and patient education and engagement in self-care for managing the symptoms. With the discrepancy in the definition of Long COVID and limited educational information in the credible public domain, neither the public nor healthcare practitioners know how best to help individuals who are infected with SARS-CoV-2 to prevent the development of Long COVID.

Currently information of patient education and self-care strategies are placed on government or relevant health organizations’ website in written form and mainly in English. There are multiple disadvantages to this practice. Firstly, access to trustworthy internet sites is not readily available or widely publicized. Secondly, the information is all in English makes it non-accessible to people who have low health literacy skills or those from non-English speaking backgrounds. Finally, and most importantly, information provision alone does not activate people to do something for their own health [44, 45]. We propose a sustainable care model that could effectively address those gaps.

### Proposed Sustainable Care Model

This proposed model is built on US, UK and Australian guidelines [12–14] that recommend early investigation, and coupled with insights from the Australian National Strategic Framework for Chronic Conditions [46] to bridge the gaps in Long COVID services through patient self-care, coordinated care and multidisciplinary collaboration with a strong focus on prevention, and patient engagement and empowerment.

**FIGURE 3:**
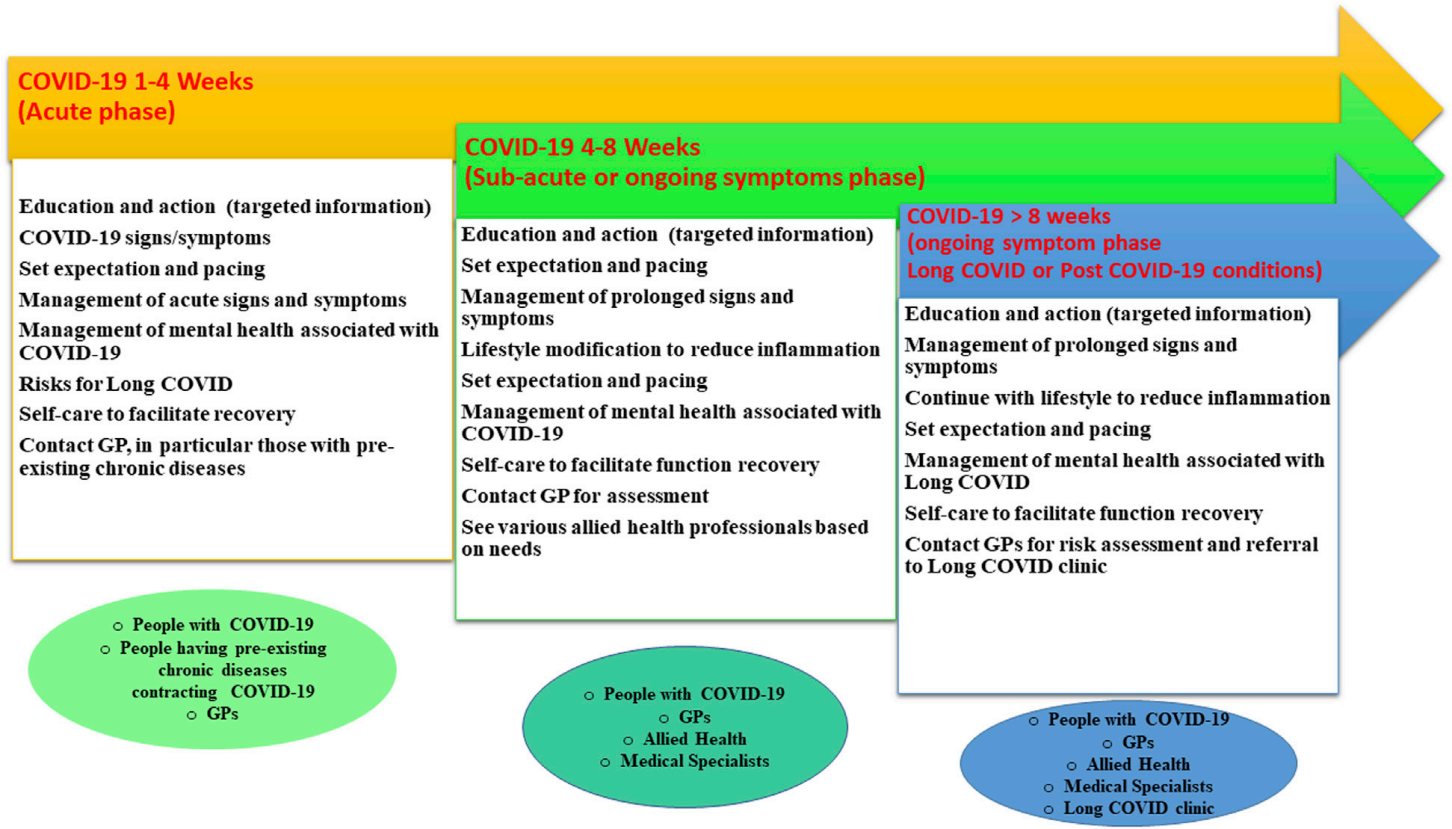
A proposed care model/pathway for Long COVID (Australia, 2023).

Our proposed Long COVID care model/pathway is outlined in Figure 3. After contracting the SARS-CoV-2 virus, individuals should be informed that the recovery period typically ranges from 10 to 120 days [47], allowing them to pace their activities accordingly. Australian data show that 90% of COVID-19 cases recovered within 8 weeks with some continuing to recover between 8 and 12 weeks [48]. In addition, people with pre-existing conditions, such as chronic fatigue, mental illness, arthritis and/or diabetes, may experience exacerbations due to COVID-19 [48, 49]. Those conditions are potential risk factors for the presentations of Long COVID [13]. This cohort is vulnerable and should be better informed and prepared. Early monitoring, intervention and prevention are essential.

Furthermore, mental health is a significant concern during the acute phase of COVID-19 and up to 3 months after infection with reports indicating up to one in three people experiencing depression or sleep problems and up to one in two facing anxiety [50]. A multicounty study demonstrates the presence of depression can be exacerbated by physical symptoms resembling COVID-19 and health information [51]. Any care model needs to include strategies to minimize the impact on mental health from the acute stage.

From 4 to 8 weeks after initial SARS-CoV-2 contraction, the sub-acute phase, individuals should contact or continue to work closely with GPs or medical specialists to investigate any ongoing symptoms of the illness [12–14, 16]. Patients are encouraged to make lifestyle changes [52, 53] aimed at reducing persistent inflammation, which is linked to Long COVID symptoms [54], and decreasing risk of developing Long COVID [52]. Self-care practices should be encouraged to facilitate recovery [12–14, 16]. Additionally, at this stage, individuals may begin seeking treatments from allied health professionals [55, 56] to help address ongoing symptoms such as shortness of breath and fatigue as well as prevent long-term complications [57].

If symptoms persist for more than 8 weeks, it is important they continue with all the components mentioned above and request their GPs or medical specialists for a risk assessment and a possible referral to a Long COVID clinic, where they can receive multidisciplinary care [12–14, 16, 47].

To promote adoption of this care model, a few critical issues, including diagnosis, workforce training, and patient education and engagement barriers, will be discussed further in the following paragraphs.

### How to Identify Long COVID and When to Treat

The diagnosis of Long COVID is based on a confirmed COVID-19 polymerase chain reaction (PCR) or rapid antigen test (RAT) positive test or suspected COVID-19 infection, and the persistence of various signs and symptoms that develop after COVID-19 where these symptoms cannot be explained by other causes or a pre-existing condition [5]. There is currently no confirmed laboratory or other forms of objective tests available globally for the diagnosis of Long COVID. So, relying on PCR or RAT and the persisting symptoms are the key strategies for the potential diagnosis of Long-COVID.

A number of guidelines advocate early review, starting within 4–12 weeks of contracting SARS-CoV-2, rather than delaying until 12 weeks as recommended by WHO. We adopt this recommendation in the proposed model. Early action and prevention of chronic conditions is prioritized in the Australian National Strategic Framework for Chronic Conditions [46] due to the substantial and growing cost of chronic diseases, both in direct medical expenses and indirect impacts. Limited data about Long COVID shows a twofold increase in direct medical costs within 12 months of contracting SARS-CoV-2 for those affected, while those without developing Long COVID experienced only a 7.5% cost increase during the same period [58]. Additionally, an analysis of New York State Workers’ Compensation Data over a 27 month period (2020–2022) during the pandemic revealed that one-third of workers’ compensation claims were attributed to Long COVID. Among claimants with Long COVID, 18% did not return to work after 12 months, and 40% of those who returned to work within 60 days continued to receive medical treatment [59].

The implementation of coordinated and multi-disciplinary programs poses however cost challenges [60], potentially burdening the healthcare system when advocating early review and intervention. So far, pulmonary rehabilitation delivered via telemedicine [61] and a tailored and multidisciplinary rehabilitation program [62] have been found to have long-term benefit for Long COVID.

We, however, could not identify any cost-effectiveness studies of treatments for Long COVID. To alleviate the potential financial burden, funding for medical investigations and Long COVID-specific programs could be sourced from various channels including a combination of resources from the government-funded programs for chronic diseases, aged care, and disability support, private health insurance, patient contributions, and philanthropic services [45]. According to Greco’s (2020) estimation, integrating enhanced self-care practices could foster sustainable healthcare outcomes [45].

### The Workforce and Workforce Training

This review finds that nearly 1/2 to 2/3 of the Australian multi-disciplinary rehabilitation services have employed allied health practitioners including physiotherapists, psychologists, exercise physiologists, dietitians and occupational therapists. Only one in 16 services included medical specialists.

A few other credentialed health professions are not included in the current services, such as practitioners of Chinese medicine, chiropractic, osteopathy, pharmacists and community nursing. These practitioners operating at the primary care level are very likely to be approached for care by impacted individuals. Not including them in potential care models may be a missed opportunity for patient education and workforce training [63, 64].

Indeed, until today, many health professionals remain uninformed about Long COVID about its diagnosis, prevention and management, thus there is an urgent need for workforce training. In addition, there is room for a multi-skilled healthcare workforce. For example, healthcare workers could be trained to conduct quick screenings for mental health symptoms and undertake centralized process for referral as required [65, 66].

### Patient Education and Engagement Barriers

Recent multi-country research showed that 90% of Long COVID cases developed from mild cases of COVID-19 [67] means that detecting the risks for Long COVID early becomes very difficult due to mild signs and symptoms. To prevent Long COVID, the most effective and immediate strategies are to educate people about the condition and provide evidence-based strategies for symptom management. This is perhaps why most guidelines encourage self-management and offer online supporting resources, which is mainly in text. This form of communication creates barriers as information does not translate into action readily.

The uptake and use of the existing Long COVID educational sites remain unclear. It will not be surprising if the resource is under-utilized. Previous research on patient activation show that given the same information, only a quarter of the population may act on the advice consistently, 40% may act on the advice initially but cannot sustain the practice, and the remaining 35% would not be able to either comprehend the information or not have the capacity to act on the advice [44, 45]. Research shows that 36% of health outcomes are due to individual behaviors, in comparison to 11% being determined by medical care [68]. It is paramount that future care models include a special section on measuring the level of patient engagement or activation so as to provide tailored information to individual needs.

### Limitations

We reviewed Australian services only and the information might not be applicable to countries not having similar healthcare services. We only accessed information in the public domain and did not survey each service for their detailed operation. To compensate for this, we invited people who have attended some of the Long COVID services to provide their feedback. Furthermore, as most of the services and guidelines are for Long COVID patients at the primary care level, this review does not address the needs of patients who were hospitalized or had been in intensive care unit due to COVID-19. We also did not discuss the government, workplace and insurance companies’ recognition of the disability associated with Long COVID [69] or the funding model of our proposed service, which are beyond the scope of this review.

### Conclusion

COVID-19 and Long COVID present a significant burden of disease and challenge to the healthcare system in Australia. In addition to Long COVID clinics, future solutions should focus on early identification and interventions that can be delivered at the primary care level by GPs and allied health practitioners. We propose a sustainable model that engages patients, with people-led self-care, further enhanced with multi-disciplinary care. COVID-19 and Long COVID present a one-in-a-century opportunity to enhance the public awareness of self-care through engaging individuals in managing the personal and societal consequence of the pandemic.
