# Supplementary material for: An Overview of Long COVID Support Services in Australia and International Clinical Guidelines, With a Proposed Care Model in a Global Context
Source: Public Health Rev. 2023 Sep 22;44:1606084. doi: 10.3389/phrs.2023.1606084 (PMC10556237; doi:10.3389/phrs.2023.1606084)
Supplement: Supplementary file 1 [file Table1.docx]

**Supplementary Appendix S1.** Search strategy (Australia, 2023)

1. Guidelines

| **Database** | **Search terms** | **Study types** | **Search date** |
| --- | --- | --- | --- |
| Google Scholar | “Long COVID guidelines"  OR  “Post COVID conditions guidelines”  AND “Post covid-19 condition”  Refined by: Review, Year 2022-2023  4044 results | Review/ Systematic Review | 10 August  2022  23 September 2022  26 January  2023  22 March  2023 |
| PDQ-Evidence | “Long COVID guidelines"  OR  “Post COVID conditions guidelines”  AND “Post covid-19 condition”  91 results | Review/ Systematic Review | 10 August  2022  23 September 2022  26 January  2023  22 March  2023 |
| WHO | “Long COVID guidelines"  OR  “Post COVID conditions guidelines”  AND “Post covid-19 condition”  1 result | Systematic Review/ Guideline | 10 August  2022  23 September 2022  26 January  2023  22 March  2023 |
| RACGP | “Long COVID guidelines"  OR  “Post COVID conditions guidelines”  AND “Post covid-19 condition”  1 result | Expert opinion/ Review/ Guideline | 10 August  2022  23 September 2022  26 January  2023  22 March  2023 |
| CDC | “Long COVID guidelines"  OR  “Post COVID conditions guidelines”  AND “Post covid-19 condition”  1 result | Systematic Review/Guideline | 10 August  2022  23 September 2022  26 January  2023  22 March  2023 |
| NIH | “Long COVID guidelines"  OR  “Post COVID conditions guidelines”  AND “Post covid-19 condition”  1 result | Review/Guideline | 10 August  2022  23 September 2022  26 January  2023  22 March  2023 |
| NICE | “Long COVID guidelines"  OR  “Post COVID conditions guidelines”  AND “Post covid-19 condition”  1 result | Systematic Review / Guideline | 10 August  2022  23 September 2022  26 January  2023  22 March  2023 |
| Public Health Agency of Canada | “Long COVID guidelines"  OR  “Post COVID conditions guidelines”  AND “Post covid-19 condition”  2 results | Guideline | 10 August  2022  23 September 2022  26 January  2023  22 March  2023 |
| Ministry of Health NZ | “Long COVID guidelines"  OR  “Post COVID conditions guidelines”  AND “Post covid-19 condition”  1 result | Review/  Guideline | 10 August  2022  23 September 2022  26 January  2023  22 March  2023 |
| Guidelines International Network (GIN) repository | “Long COVID guidelines"  OR  “Post COVID conditions guidelines”  AND “Post covid-19 condition  3 results | Systematic Review/  Guideline | 12 July  2023 |
