# Supplementary material for: An Overview of Long COVID Support Services in Australia and International Clinical Guidelines, With a Proposed Care Model in a Global Context
Source: Public Health Rev. 2023 Sep 22;44:1606084. doi: 10.3389/phrs.2023.1606084 (PMC10556237; doi:10.3389/phrs.2023.1606084)
Supplement: Supplementary file 2 [file Table2.docx]

**Supplementary Appendix S2.** A summary of international and Australian national guidelines on the management of Long COVID (Australia, 2023)

| **References** | **Screening/**  **Assessment** | **Care principles** | **Care models** | **Patient education** | **Red flags** | **Comments** |
| --- | --- | --- | --- | --- | --- | --- |
| [5]  WHO | 1. WHO Post COVID-19 CRF*,  2. The Post COVID-19 Functional Status scale (PCFS). | Early referral (at or before 12 weeks),  Multidisciplinary rehabilitation teams, Care continuity and coordination, People-centred care. | Interdisciplinary work,  Patient engagement, Rehabilitation requirement assessment, Outcome measurement, Patient education, Guided self-management, Recommendations for 11 common symptoms and dysfunctions,  Hybrid delivery method. | Consistently recommended in 10 of 11 key symptoms and functional impairments. | Exertional desaturation. Cardiac impairment.  Post-exertional  symptom exacerbation (PESE) needs modified intervention. | The guidelines build on systematic reviews,  provide a framework for the rehabilitation program,  recommend for each common symptom and dysfunction,  comprehensive information.  However, they are lacking step-by-step approaches to skills training interactive or visual information for education and skills building. Didn’t mention if individuals who have pre-existing conditions and experience worsened symptoms after contracting COVID-19 should be included. |
| [12]  Australia | Patient assessment:  1.History of acute COVID-19  2.Nature and severity of previous and current symptoms  3. Duration of symptoms since the initial diagnosis of COVID-19  4. Medical history of other health issues,  5. Exacerbation of any pre-existing conditions  6. Mental health | Early referral to allied health,  Holistic & person-centered approach:  Taking a comprehensive clinical history, Assessing physical, cognitive, psychological and psychiatric symptoms, as well as functional abilities. | GP coordinated multidisciplinary care with patient education,  Patient collaboration, Connecting patients with social services and peers,  Rule out red flags. | Guide available for self-managing post COVID-19 conditions,  Encourage patients to fill in a symptom diary every few days. | Severe, new onset, or worsening breathlessness or hypoxia,  Syncope,  Unexplained chest pain, palpitations or arrhythmias  New delirium, or focal neurological symptoms | The guidelines complement the WHO guideline.  GP care plan is outlined throughout the COVID-19 journey. This document focus on post- acute COVID recovery and helps coordinate the care for Long COVID. The guidelines have also developed patient resources for self-management. |
| [13]  US | Comprehensive physical examination (standard vital signs, body mass index, ambulatory pulse-oximetry, orthostatic vital signs),  Medical history,  Laboratory testing  (Blood count, electrolytes, and renal function, liver function, Inflammatory markers, etc.),  Functional status or quality of life,  Respiratory conditions,  Neurologic conditions,  Psychiatric conditions,  Exercise capacity,  Balance and fall risk. | Initial evaluation and supportive care/referral between 4-12 weeks after diagnosis of COVID-19,  Holistic patient centred approach. | Use the ICD-10 code** for Long COVID condition in US health records  Six guiding principles to a trauma-Informed approach,  Partnership between specialists and rehabilitation services,  Peer support via social service connection. | Simple resources available for self-management,  Encourages patient symptom diaries and calendars,  Provide links to support groups. | Pulmonary embolism, myocardial infarction, pericarditis with effusion, stroke,  renal failure | The guidelines recommend early assessment and management.  They are the first one mentioned about trauma-informed approaches (6 principles), also have detailed physical examination suggestions.  The guidelines have discussed the different presentations of Long COVID conditions, such as an exacerbation of pre-existing condition.  Also, there are advice on self-managing physical and mental symptoms post COVID-19. |
| [14, 16]  UK | Assess:  Physical,  Cognitive,  Psychological, Psychiatric, symptoms, Functional  Abilities,  Comprehensive clinical history (exacerbation of any pre-existing conditions),  Blood tests (full blood count, kidney and liver function, C reactive protein, thyroid function etc.),  Chest radiograph,  Exercise tolerance test. | Holistic assessment,  Referral to  multidisciplinary care from 4 weeks post the start of the acute COVID-19,  Rule out life-threatening complications. | Offer coordinated primary care,  community, rehabilitation, and mental health services,  Provide referral to relevant multidisciplinary assessment  service,  Or to specialist care,  Education for self-management,  Follow up and continuity of care. | New or ongoing symptoms after acute COVID-19, Expectations for recovery,  Recommendations for self-management,  When to seek help from healthcare professional,  Symptom  diaries and symptom tracking apps. | Severe hypoxaemia,  Oxygen desaturation on exercise,  Severe lung disease,  Cardiac chest pain,  Multisystem inflammatory syndrome (in children). | Summarized the NICE, SIGN, and RCGP rapid guidelines.  Comprehensive and has the care for the journey, from post COVID acute stage to Long COVID; focus on providing care, patient education and self-management.  Linked to NHS website; recommendation for service organization; one-stop clinic model of multidisciplinary clinics; integrated, multidisciplinary service; seamless care; and referral pathway. |
| [15]  New Zealand | A comprehensive medical assessment, using a Long COVID symptom map. | Holistic care and assessment,  Referral to  Multidisciplinary rehabilitation team and specialist care,  Rule out life-threatening complications. | Assigned an ICD-10-AM code *** for Long COVID  Personalised management plan,  Multidisciplinary and specialist care,  Virtual assistance,  Accommodate to individual’s needs,  Self-management. | Links and helpline numbers for mental health support,  Links for Long COVID related symptom management,  Peer support,  Resources for children and young people with Long COVID,  Care plans and action plans. | Heart failure  Pulmonary embolism  Acute coronary syndrome  Post-exertional symptom exacerbation (PESE)  Myocarditis  Chest pain  Tightness, worsening or increasing palpitations, dyspnoea, desaturation in exertion  Coagulation dysfunction  Neurological disorder | The guidelines offer care pathway recommendations tailored to various population groups, such as Māori, Pacific, children, disadvantaged individuals, and the elderly.  In addition, the guidelines provide suggestions for vocational rehabilitation.  However, there is no timeframe specified for making a referral, |

* [WHO Post COVID-19 CRF screening tool](https://apps.who.int/iris/bitstream/handle/10665/345299/WHO-2019-nCoV-Post_COVID-19_CRF-2021.2-eng.pdf)

** [An ICD-10 code for Long COVID condition, page 30](https://www.cdc.gov/nchs/data/icd/10cmguidelines-FY2022-7-2022-508.pdf)

*** [ICD-10-AM code for Post COVID-19 syndromes used in New Zealand](https://www.tewhatuora.govt.nz/for-the-health-sector/covid-19-information-for-health-professionals/recording-covid-19)
