# Supplementary material for: An Overview of Long COVID Support Services in Australia and International Clinical Guidelines, With a Proposed Care Model in a Global Context
Source: Public Health Rev. 2023 Sep 22;44:1606084. doi: 10.3389/phrs.2023.1606084 (PMC10556237; doi:10.3389/phrs.2023.1606084)
Supplement: Supplementary file 3 [file Table3.docx]

**Supplementary Appendix S3.** Distribution (%) of health professionals in the rehabilitation team across the 16 identified Long COVID services in Australia (Australia, 2023)

| **Health professionals** | **Distribution**  **(% of Long COVID Services)** |
| --- | --- |
| Physiotherapist | 63 |
| Psychologist | 63 |
| Exercise physiologist | 44 |
| Occupational therapist | 38 |
| Dietitian | 38 |
| Respiratory physician | 31 |
| Rehabilitation physician | 31 |
| Social worker | 25 |
| Speech therapist | 19 |
| GP | 19 |
| Nurse | 19 |
| General Medicine physician | 13 |
| Sleep physician | 13 |
| Cardiologist | 13 |
| Music therapist | 13 |
| Neurologist | 6 |
| Liaison psychiatrist | 6 |
| Pain specialist | 6 |
